# Protective Effect of Biobran/MGN-3, an Arabinoxylan from Rice Bran, Against the Cytotoxic Effects of Polyethylene Nanoplastics in Normal Mouse Hepatocytes: An In Vitro and In Silico Study

**DOI:** 10.3390/nu17121993

**Published:** 2025-06-13

**Authors:** Heba Allah M. Elbaghdady, Rasha M. Allam, Mahmoud I. M. Darwish, Maha O. Hammad, Hewida H. Fadel, Mamdooh H. Ghoneum

**Affiliations:** 1 Department of Zoology, Faculty of Science, Mansoura University, Mansoura 35516, Egypt; dr_hebagad1982@yahoo.com; 2 Pharmacology Department, National Research Centre (NRC), Cairo 12622, Egypt; 3 Department of Biochemistry and Molecular Biology, Faculty of Veterinary Medicine, Zagazig University, Zagazig 44519, Egypt; 4 Department of Medical Biochemistry and Molecular Biology, Faculty of Medicine, Mansoura University, Mansoura 35516, Egypt; maha_osman@mans.edu.eg; 5 Department of Medical Laboratory Technology, Faculty of Applied Health Sciences Technology, Pharos University in Alexandria, Alexandria 21568, Egypt; hewida.fadel@pua.edu.eg; 6 Department of Surgery, Charles Drew University of Medicine and Science, Los Angeles, CA 90059, USA; 7 Department of Surgery, University of California Los Angeles, Los Angeles, CA 90095, USA

**Keywords:** Biobran, MGN-3, polyethylene nanoplastics, hepatocytes, apoptosis, G2/M cell arrest

## Abstract

Background: Plastic is one of the most versatile and widely used materials, but the environmental accumulation of nanoplastics (NPs) poses a risk to human health. Preclinical studies have verified that the liver is one of the main organs susceptible to NPs. Biobran/MGN-3, an arabinoxylan from rice bran, has been shown to have hepatoprotective effects; here, we show Biobran’s ability to alleviate polyethylene nanoplastics (PE-NPs)-induced liver cell toxicity by reversing apoptosis and restoring G2/M cell arrest in mouse liver cells (BNL CL.2). Methods: Toxicological effects were measured using the sulforhodamine B (SRB) assay for cell viability and flow cytometry for cell cycle analysis and apoptosis. An in silico study was also used to demonstrate the docking of PE-NPs to pro-inflammatory mediator proteins (IL-6R, IL-17R, CD41/CD61, CD47/SIRP), cell cycle regulators (BCL-2, c-Myc), as well as serine carboxypeptidase, which is an active ingredient of Biobran. Results: Exposing liver cells to PE-NPs caused a significant decrease in cell viability, with an IC50 value of 334.9 ± 2.7 µg/mL. Co-treatment with Biobran restored cell viability to normal levels, preserving 85% viability at the highest concentration of PE-NPs. Additionally, total cell death observed after exposure to PE-NPs was reduced by 2.4-fold with Biobran co-treatment. The G2/M arrest and subsequent cell death (pre-G0 phase) induced by PE-NPs were normalized after combined treatment. The in silico study revealed that Biobran blocks the nucleophilic centers of PE-NPs, preventing their interaction with pro-inflammatory mediators and cell cycle regulators. Conclusions: These findings highlight the potential use of Biobran as a hepatoprotector against NP toxicity.

## 1. Introduction

Plastic pollution is a serious ecological issue that threatens global environmental security and is receiving greater attention from governments, the scientific community, and the general public [1]. A variety of factors, such as wastewater discharge, littering, unlawful dumping, and the use of plastic for packaging and equipment for aquatic and human activities, allow plastic debris to enter ecosystems. The most commonly used and produced plastic polymers for human consumption are polyethylene (PE), polyethylene terephthalate (PET), polypropylene (PP), polyvinyl chloride (PVC), polyamide (PA), and polystyrene (PS); these can all be found in the sea [2]. Plastic pollutants are categorized by size into macroplastics, mesoplastics, microplastics, and nanoplastics (NPs). Organisms have the ability to consume these various forms and pass them along food chains. Plastics can break down into a broad variety of sizes, including, in the case of biological degradation, micro- (<5 mm) and nano-sized (<100 nm) particles. Abrasion and exposure to UV radiation also occur naturally. All ecosystems may be at risk from pollution caused by micro- and nanoplastics [3]. Plastic particles may be consumed by creatures found near the base of the food chain because of their small size, and they can then be fed to higher consumers [4]. Several investigations have shown that tissue damage or even death can occur to aquatic organisms when they swallow plastic particles of different sizes [5]. Therefore, it has recently been acknowledged that water-related plastic pollution and its possible effects on aquatic life are significant concerns for both ecosystem functioning and society [6]. Furthermore, NPs can enter cells and come into contact with more complex biological fluids due to their capacity to penetrate cell membranes [7]. Prior studies have demonstrated that consuming NPs may cause oxidative stress, injury to tissues, and disruption of fish-feeding behavior and metabolism. Subsequently, NPs interact with proteins in more ways, affecting various biological processes in organisms, including blood coagulation, immune system function, lipid metabolism, and reproduction [8].

NPs constitute a wide range of xenobiotics that are toxic to the liver, an organ whose main function is the detoxification of xenobiotics and endogenous compounds. Negative effects on the pro-inflammatory signal pathways that include IL-6/IL-6R and IL-17/IL-17R can lead to hepatitis, steatohepatitis, cirrhosis, and liver cancer [9,10]. It has further been demonstrated that the upregulation of integrins such as αIIbβ3 (CD41/CD61) plays a role in the progression of liver fibrosis, blood coagulation, platelet aggregation, and thrombosis formation [11,12]. Blockading the integrin-associated protein CD47/SIRP can reduce hepatic fibrosis [13]. Cytokines are also related to BCL-2, as it has been shown that overexpression of IL-6 and IL-6R enhances cell proliferation by increasing BCL-2 expression and thereby causes the proliferation/apoptosis balance to shift toward neoplastic transformation [14]. Long-term exposure to xenobiotics such as plastics can result in chronic inflammation, a condition whose role in cancer progression is well established [15]. There is strong evidence indicating an anti-apoptotic action of BCL-2. A previous study indicated that overexpression of BCL-2 leads to a poor prognosis of hepatocellular carcinoma [16]. c-MYC signaling is another pathway and one of the most frequently proliferative signals in hepatocarcinogenesis [17], and one that has been indicated in plastic-induced carcinogenesis [18].

A variety of natural food products have been recently investigated for their hepatoprotective effects. Cordycepin, a natural product isolated from traditional Chinese medicine, has been shown in mouse studies to attenuate lipid accumulation, inflammation, and lipotoxicity in hepatocytes subjected to metabolic stress [19]. Rosemary essential oil (REO), a well-known antioxidant product in the food industry, has been evaluated for its protective effect on carbon tetrachloride (CCl_4_)-induced liver injury in rats, with results showing that REO decreased AST and ALT activities up to 2-fold and prevented CCl_4_-induced increase of lipid peroxidation in liver homogenates [20]. Theanine (LTA) and epigallocatechin gallate (EGCG) in tea exert antioxidant and hepatoprotective effects, and a recent study showed that their combined use alleviated alcoholic fatty liver disease by modulating lipid metabolism and ameliorating oxidative stress, indicating their potential as natural active ingredients in anti-alcoholic fatty liver food products [21]. Nerol, a monoterpene, has been shown to protect against paracetamol-induced liver toxicity in a rat model [22]. Shibi tea, a non-*Camellia* tea prepared from the dried leaves of *Adinandra nitida*, a plant with a high flavonoid concentration, protected mice against CCl_4_-induced liver injury via mechanisms that included antioxidative stress, anti-inflammatory, and anti-apoptotic effects [23]. Finally, hepatocarcinogenesis in rats and hepatocellular carcinoma in human patients have been shown to be counteracted by the natural rice bran product Biobran [24,25], in part through the induction of apoptosis, inhibition of inflammation, and suppression of cancer cell proliferation.

We hypothesized that the natural Biobran product might similarly be able to counteract the negative effects of NPs on the liver. Biobran is a denatured hemicellulose that is obtained by reacting rice bran hemicellulose with multiple carbohydrate-hydrolyzing enzymes from Shiitake mushrooms [26], and it has been investigated over the last 30 years for its immunomodulatory effects and potential as an anticancer agent [27,28]. In vivo studies have shown that Biobran induces tumor regression and anticancer effects for a range of cancers, including gastric cancer [29], Ehrlich carcinoma [30], neuroblastoma [31], and multiple myeloma [32]. In vivo studies have also shown that Biobran activates immune cells with anticancer activity known as natural killer (NK) cells [27,31,33,34]. In addition, Biobran has been shown to have positive effects against viral infections such as HIV [26], hepatitis C [35], influenza-like illnesses (ILI) in elderly subjects [36], and severe acute respiratory syndrome coronavirus 2 (SARS-CoV-2) [37], with anti-COVID-19 effects and boosted immunity in human subjects [38].

This study investigates our hypothesis on the positive effect of Biobran in counteracting NP-induced liver toxicity by measuring the toxicological effects of polyethylene nanoplastics (PE-NPs) on mouse liver cells (BNL CL.2). Flow cytometry is used to analyze the cell cycle and apoptosis, and the SRB assay is used to evaluate cell viability. To investigate the mechanism of action, we also carried out an in silico study to interpret the effect of PE-NPs on cell response and cell cycle, as well as the possible protective effect of Biobran via molecular docking.

## 2. Materials & Methods

### 2.1. Sample Preparation for Biological Assays

Biobran was provided by Daiwa Pharmaceutical Co., Ltd., Tokyo, Japan. Polyethylene nanoplastics (PE-NPs) were commercial grade MPP-635XF (CAS: 9002-88-4), purchased from Micro Powders Inc. (Tarrytown, NY, USA). PE-NPs consisted of raw white powder with a mean particle size ranging from 4.0 to 6.0 μm. Biobran and PE-NPs were dissolved in PBS using probe-sonication at a 10 mg/mL stock solution.

### 2.2. Characterization of PE-NPs

**Figure 1:**
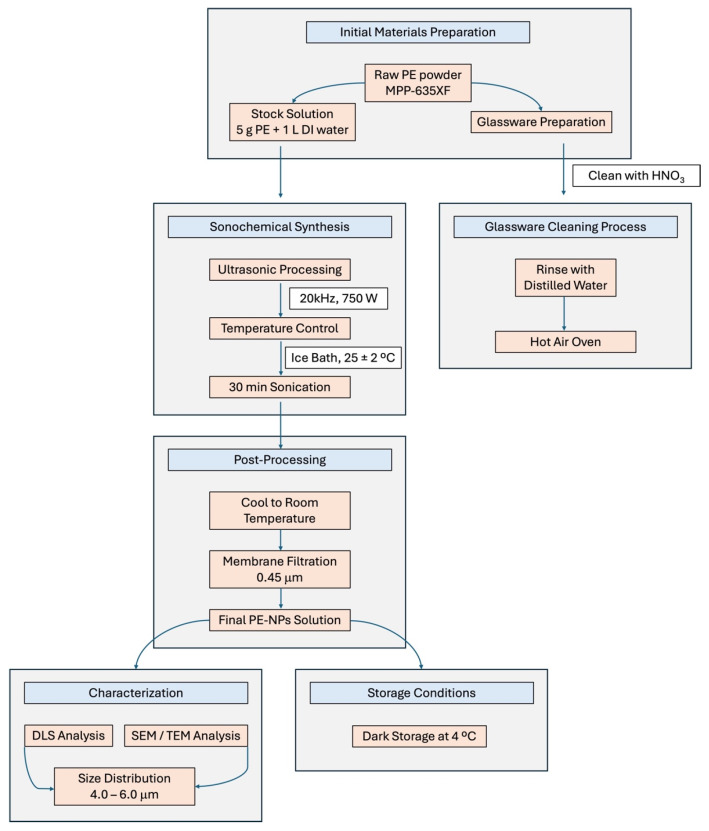
Flow diagram illustrating the synthesis process of PE-NPs via the sonochemical method. The process is divided into four main stages: (1) initial materials preparation, showing raw PE powder and stock solution preparation; (2) sonochemical synthesis, detailing ultrasonic processing conditions; (3) post-processing, including membrane filtration; and (4) final stages comprising characterization and storage conditions.

All glass items used in the experiments were thoroughly cleaned with diluted nitric acid (HNO_3_) and distilled water, followed by drying in a hot air oven to ensure contamination-free conditions. The synthesis of PE-NPs was performed using the sonochemical method (see Figure 1). Initially, a stock solution was prepared by dissolving 5 g of PE-NPs in 1 L of distilled water at room temperature, following the manufacturer’s specifications, to achieve a concentration of 5 g/L. The solution was then transferred to a jacketed glass reactor maintained at 25 ± 2 °C using an external ice bath. Sonication was carried out using a high-intensity ultrasonic processor operating at a frequency of 20 kHz and a power output of 750 W. The sonication process was conducted in pulse mode (5 s on/5 s off) for a total duration of 30 min to prevent excessive heat generation and ensure uniform particle size distribution. Post-sonication, the suspension was allowed to cool to room temperature and was filtered through a 0.45 μm membrane filter to remove any large aggregates. The filtered suspension was stored in dark conditions at 4 °C to maintain stability. Before each experimental use, the stock solutions were thoroughly shaken to ensure homogeneous dispersion of the nanoplastics. All experiments were conducted using deionized water to maintain consistency and prevent interference from impurities. The synthesized PE-NPs demonstrated consistent size distribution within the target range of 4.0–6.0 μm, confirming the effectiveness of the sonochemical synthesis method.

**Figure 2:**
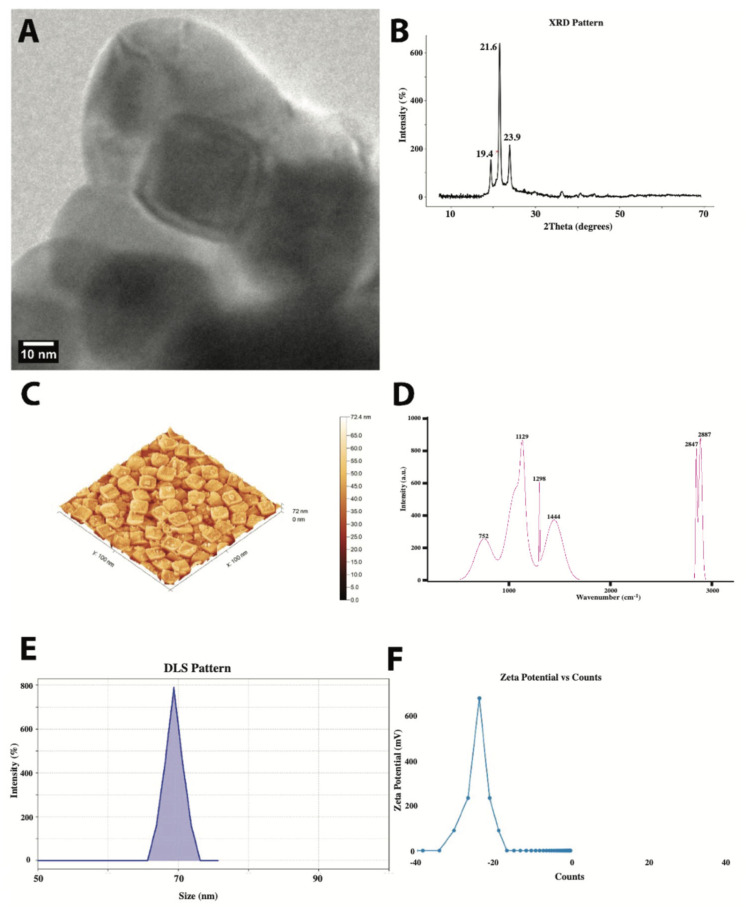
Multi-technique characterization of PE-NPs: (**A**) TEM image showing spheroidal morphology with some aggregation (scale bar 10 nm); (**B**) XRD pattern displaying characteristic orthorhombic polyethylene peaks at 19.4°, 21.6°, and 23.9°; (**C**) 3D AFM topography revealing quasi-cubic surface structures with height variations up to 72.4 nm; (**D**) Raman spectrum with distinctive polyethylene vibrational modes at 752, 1129, 1298, 1444, 2847, and 2887 cm^−1^; (**E**) DLS analysis showing narrow size distribution centered at ~70 nm; and (**F**) Zeta potential distribution with peak at approximately −25 mV.

Particle size distribution and morphology were characterized using a variety of measurement techniques. Figure 2A shows a transmission electron microscope image that demonstrates the predominantly spheroidal morphology of PE-NPs with some degree of faceting. The particles had well-defined boundaries and relatively uniform electron density distribution, indicating a homogeneous internal structure. Quantitative analysis showed an average particle size of 45 ± 8 nm, with individual particles ranging from 30 to 65 nm in diameter. The particles displayed a tendency to form loose aggregates while maintaining their individual morphological integrity, with distinct boundaries even within these assemblies. X-ray diffraction of PE-NPs (Figure 2B) showed multiple well-defined peaks characteristic of semi-crystalline polyethylene, with a crystallinity index indicating a crystallinity degree of approximately 72%. Atomic force microscopy (Figure 2C) revealed a characteristic surface pattern of closely packed, quasi-cubic structures with a relatively uniform size distribution. The measured surface area of 397,693 nm^2^ compared to the projected area of 10,000 nm^2^ indicates significant surface development, with a surface area ratio of approximately 40. The Raman spectrum of PE-NPs (Figure 2D) exhibited distinct peaks, with the high-frequency region showing two notable peaks at 2847 and 2887 cm^−1^, assigned to the symmetric and asymmetric CH_2_ stretching vibrations, respectively. The lower frequency peak at approximately 752 cm^−1^ corresponds to the CH_2_ rocking mode. Dynamic light scattering (Figure 2E) revealed a well-defined unimodal size distribution with a mean hydrodynamic diameter of approximately 70 nm. The measured polydispersity index (PDI) of 0.082 confirmed the high monodispersity of the sample. The narrow peak width of about 12.4 nm suggested excellent control over the particle size during synthesis; the sample was free from significant aggregation or the presence of multiple particle populations. Zeta potential measurements (Figure 2F) showed a well-defined single and narrow peak width, indicating a uniform surface charge distribution across the particle population and a moderately stable range for colloidal systems.

### 2.3. Cell Culture

Murine liver cells (BNL CL.2) were obtained from Nawah Scientific Inc. (Cairo, Egypt). Cells were maintained in Dulbecco’s Modified Eagle Medium (DMEM, Gibco, Grand Island, NY, USA) supplemented with 100 μg/mL of streptomycin (Lonza GmbH, Köln, Germany), 100 units/mL of penicillin (Lonza GmbH, Köln, Germany), and 10% fetal bovine serum (FBS; Gibco, NY, USA). BNL was cultivated in a condition of 37 °C and a humidified atmosphere containing 5% CO_2_.

### 2.4. Biological Studies

#### 2.4.1. Cytotoxicity Evaluation by SRB Assay

The sulforhodamine B (SRB) assay was performed to assess the toxicity of PE-NPs, Biobran, and their combination on the viability of liver cells (BNL CL.2). In 96-well plates, cells were seeded at a density of 1 × 10^3^ cells/well using different log and half-log concentrations ranging (0.1 to 300 µg/mL) for 72 h. Later, the culture medium was aspirated, and cells were fixed for 1 h at 4 °C with 150 μL of 10% trichloroacetic acid (TCA) (Merck, Warsaw, Poland). This was followed by washing the cells five times with distilled water. Then, 70 μL of SRB solution (Sigma-Aldrich, Burlington, MA, USA) (0.4% *w*/*v*) was added and incubated for 10 min at room temperature in the dark. Cells were washed three times with 1% acetic acid (Chem-Lab) and allowed to air-dry. Then, 150 μL of Tris pH 10.5 (Chem-Lab) (10 mM) was added, and the absorbance was measured at 540 nm using a BMG LABTECH^®^-FLUOstar Omega microplate reader (Ortenberg, Germany). Half-maximal inhibitory concentration (IC50) values were calculated for each experiment using GraphPad Prism 6 software. IC50 values were reported as mean ± SD [39].

#### 2.4.2. Cell Cycle Analysis—Cytotoxicity Evaluation by Flow Cytometry

The cell cycle phases of liver BNL CL.2 cells were analyzed using flow cytometry after treatment with Biobran, PE-NPs, and their combination. In six-well plates, cells were seeded at a density of 1 × 10^4^ cells/well and incubated for 24 h. Then, the cells were treated for 48 h. After that, the cells were trypsinization, pelleted, washed twice, and resuspended with 1 mL of phosphate-buffered saline (PBS, Lonza GmbH, Köln, Germany). Then, the cells were fixed with 60% ethanol at 4 °C for a minimum of 2 h. After washing with PBS, the pellets were treated with RNase (Sigma-Aldrich, Louis and Burlington, MA, USA) and stained with propidium iodide (PI, Sigma-Aldrich) at 37 °C in the dark for 30 min. The cellular DNA content was determined using the ACEA NovoCyte™ flow cytometer (ACEA Biosciences Inc., San Diego, CA, USA). The findings were evaluated using the ACEA NovoExpress™ software (version 1.6.3, ACEA Biosciences Inc., San Diego, CA, USA) [40].

#### 2.4.3. Annexin V/PI Apoptotic Assay

The impact of Biobran, PE-NPs, and their combination on the apoptotic/necrotic cell death of liver BNL CL.2 cells was investigated using the Annexin V-FITC Apoptosis Staining/Detection kit (ab14085; Abcam, Cambridge, MA, USA). In brief, cells were planted in six-well plates at a density of 1 × 10^4^ cells/well for 24 h and exposed to treatment for another 48 h with the previously determined IC50 values. After that, the cells were trypsinized and washed twice with PBS. The cells were then resuspended in 500 μL of 1X binding buffer and stained with Annexin V-FITC and PI for 30 min at room temperature in the dark [41].

### 2.5. In Silico Study

To explore the efficacy of Biobran against PE-NP-induced cell damage, we carried out docking of serine carboxypeptidase (pdb:3sc2)—the active ingredient of Biobran—to PE-NPs, as well as docking of PE-NPs to pro-inflammatory mediators IL-6R (pdb:1n26), IL-17R (pdb:5n9b), (pdb:3fcs), and CD47/SIRP (pdb:2jjs), and to the oncogenic proteins c-MYC (pdb:6m75), BCL-2 isoform I (pdb:1g5m), and isoform II (pdb:1gjh). We used Avogadro’s energy for molecular optimization, Spdbv for the energy optimization of proteins, Discovery Studio 2021 for visualization of the ligand–receptor, and iGEMDOCK for docking. Additionally, we used the epitope predictor to predict the B-cell epitopes on the examined proteins [42].

### 2.6. Statistical Analysis

Data were analyzed using GraphPad Prism 6 software. Results are presented as mean ± standard deviation (SD) from at least three independent experiments. Statistical significance was determined using one-way analysis of variance (ANOVA) followed by Tukey’s post hoc test for multiple comparisons. Differences were considered statistically significant at *p* < 0.05.

For the SRB cytotoxicity assay, IC50 values were calculated using non-linear regression analysis. Cell cycle and apoptosis data from flow cytometry experiments were analyzed using ACEA NovoExpress™ software. Percentages of cells in different cell cycle phases and apoptotic/necrotic populations were compared between treatment groups using one-way ANOVA with Tukey’s post hoc test. All experiments were performed in triplicate and repeated at least three times independently to ensure reproducibility of results. Sample sizes were determined based on preliminary experiments to achieve adequate statistical power.

## 3. Results

### 3.1. Toxicological Assessment

**Figure 3:**
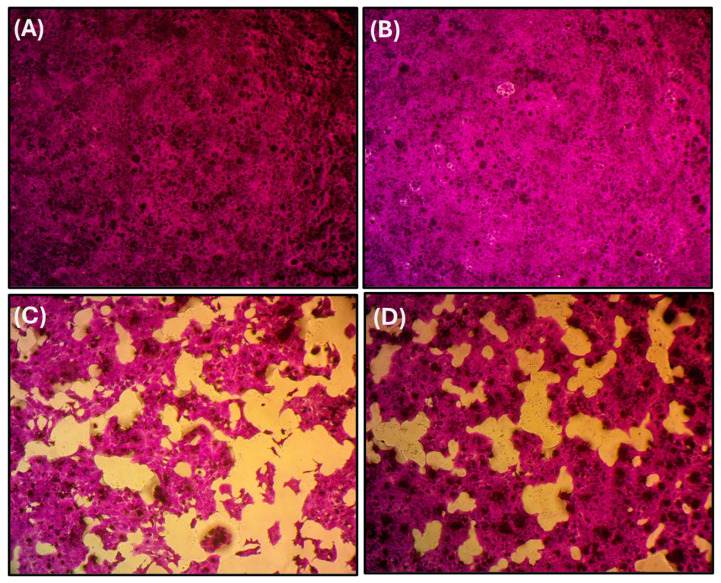
Inverted light microscopic (ILM) photographs displaying changes in cell count throughout the cytotoxicity experiment in the BNL CL.2 cells after 72 h of different treatments. The photos show the normal architecture of the untreated control (**A**), treatment with Biobran (**B**), PE-NPs (**C**), or their combination (**D**). After removing the media, the cells were fixed with TCA, stained with SRB dye, and visualized under an ILM at 100×.

**Figure 4:**
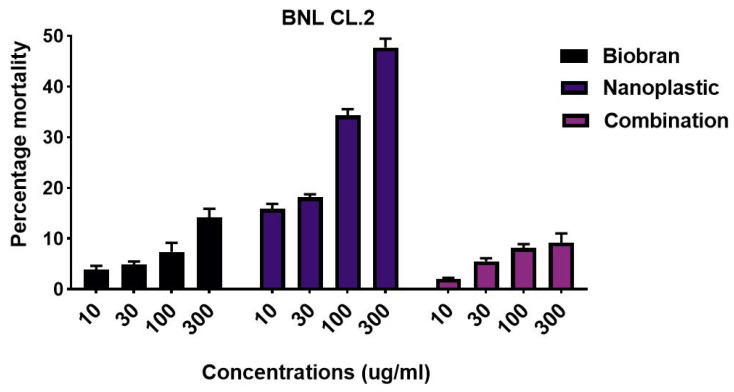
SRB assay evaluating cytotoxic effects of PE-NPs, Biobran, and their combination in normal liver cells (BNL CL.2) after treatment for 72 h. Data are presented as mean ± SD.

To assess the cytotoxic effect of PE-NPs, liver cells were exposed to different PE-NP concentrations of 0.3, 1, 3, 10, 30, 100, 300, and 1000 µg/mL for 72 h, with an IC50 of 411.01 µg/mL. The potential toxicity of PE-NPs and the possible hepatoprotective effect of Biobran were assessed after 72 h of treatment on liver cell viability via the SRB assay (Figure 3 and Figure 4). PE-NPs showed a dose-dependent decrease in cell viability of BNL CL.2 cells, with a dramatic reduction observed at 300 µg/mL, resulting in approximately 52% viability with an IC50 value of 334.9 ± 2.7 µg/mL. Reciprocally, exposure of BNL CL.2 cells to all treatment concentrations of Biobran ranging from 0.1 to 300 µg/mL did not show a significant reduction in cell viability compared to control cells, even at the highest concentration of 300 µg/mL yielding only a 14% decrease in viability, indicating a mild effect on this cell line and confirming its safety. Interestingly, co-treatment of PE-NPs with Biobran at a ratio of 1:1 significantly re-increased the viability of cells compared to PE-NPs alone, reaching about 90% at 300 µg/mL. After combining PE-NPs (300 µg/mL) with Biobran (340 µg/mL), the IC50 of the combination increased to 824.89 µg/mL compared to 411.01 µg/mL for PE-NPs alone. These findings indicate for the first time that Biobran can ameliorate the cytotoxic effect of PE-NPs on liver cells.

### 3.2. Effect on Cell Cycle

**Figure 5:**
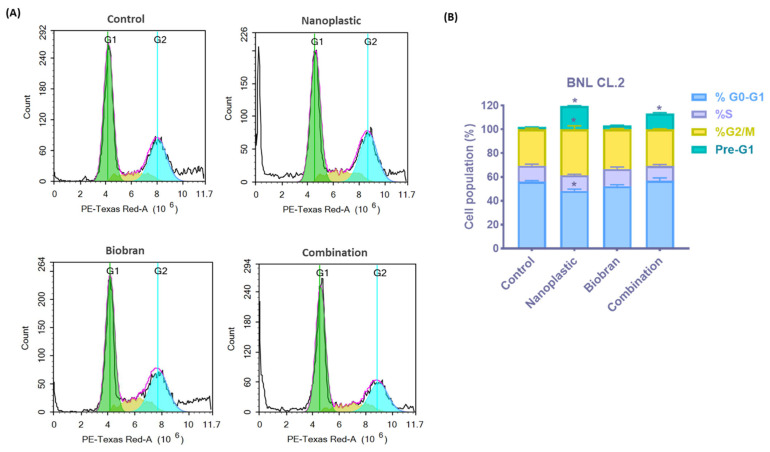
(**A**) Cell cycle distribution in BNL CL.2 cells after treatment with PE-NPs, Biobran, and their combination for 48 h was determined using DNA cytometry analysis compared with control (untreated cells). (**B**) The percentage of cells in all cell cycle phases, including the pre-G1 phase, was plotted as a bar graph of mean ± SD; n = 3. * Statistically significant from control at *p* < 0.05.

The effects of PE-NPs and Biobran on the different phases of the cell cycle in normal liver cells were analyzed by flow cytometry using PI-stained cells, as shown in Figure 5. The percentage of hypodiploid cells in the sub-G1 phase, indicative of cell death, markedly increased in the PE-NPs-treated cells to 19.51 ± 0.3%, compared to 1.92 ± 0.1% in the control group. In contrast, Biobran treatment resulted in only 3.28 ± 0.3% of cells in the sub-G1 phase, while combined treatment with PE-NPs reduced the hypodiploid cells in the sub-G1 to 13.2 ± 0.8%, which was still less than that observed with PE-NPs alone.

No significant differences were observed across all treatment groups in the DNA synthesis phase (S phase) compared to the control group. Likewise, no changes were observed in the G0/G1 phase (proliferating fraction) and G2/M phase (mitotic fraction), except for the treatment of PE-NPs. This treatment showed a significant reduction in the percentage of cells in the G0/G1 phase from 56.19 ± 0.9% to 48.4 ± 1.5%, concomitantly with an increase in the G2/M phase from 30.56 ± 0.9% to 39.67 ± 2.4%, indicating G2/M arrest and PE-NPs’ disruption of mitosis. These data verify the results from the viability assay, further demonstrating that Biobran can mitigate the toxicity of nanoplastics on liver cells.

The anti-proliferative effect of PE-NPs was assessed by flow cytometry. FACS profiles showed that nanoplastic arrested most of the cells in the G0/G1 phase (non-proliferating cells). Therefore, most of the cells (52.22%) failed to go to the subsequent phases of mitotic division (Figure 5). A very low percentage of cells (3.34%) entered sub-G1. After treatment of BNL cells with a combination of PE-NPs (300 µg/mL) and Biobran (340 µg/mL), we noticed that the cell cycle of BNL cells improved. The percentage of BNL in the G0/G1 phase was 50.82%, and the percentage of cells that entered the sub-G1 phase was 14.38% (Figure 5).

### 3.3. Effect on Cell Death Modes (Apoptosis/Necrosis)

**Figure 6:**
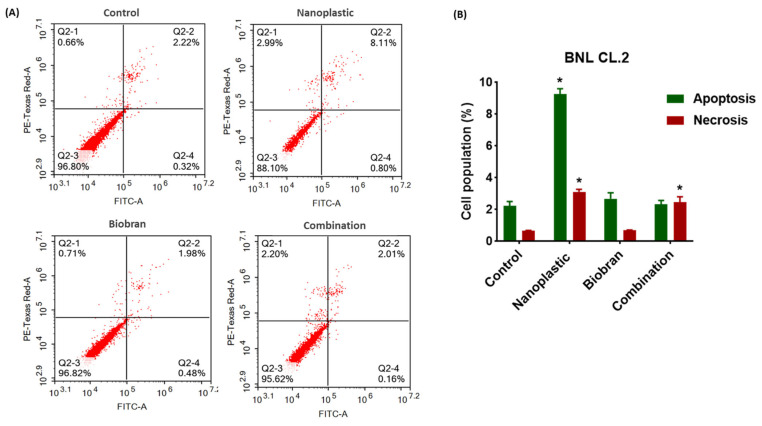
(**A**) Apoptosis/necrosis assessment by flow cytometry in BNL CL.2 cells with PE-NPs, Biobran, and their combination for 48 h and stained with PI/Annexin V-FITC. (**B**) Different cell populations at apoptosis and necrosis were plotted as percentages of total events. Data are presented as mean ± SD; n = 3. * Statistically significant from control at *p* < 0.05.

To specify the cell death mode, that is, whether apoptosis and/or necrosis were induced by PE-NPs in BNL CL.2 cells, and to further explore the modulation of cell cycle phases and cell death detected in cell cycle analysis, flow cytometry was coupled with Annexin-V-FITC/PI staining. The results are shown in Figure 6. PE-NPs triggered both apoptotic and necrotic cell death, with apoptotic cell death being predominant compared to untreated cells (Figure 6A,B). The levels of liver cell apoptosis (including early apoptosis and late apoptosis) after exposure to PE-NPs increased to 9.265 ± 0.34% compared to 2.24 ± 0.26% for untreated cells, while levels of liver cell necrosis increased from 0.66 ± 0.03% to 3.11 ± 0.16%, with total cell death reaching nearly 12% of cells. Meanwhile, treatment with Biobran alone did not induce any change in apoptotic and necrotic cell death compared to control cells. Combining Biobran with PE-NPs markedly reduced total cell death to nearly 5%, with 2.34 ± 0.23% apoptosis and 2.45 ± 0.35% necrosis, representing a significant improvement compared to the 12% cell death induced by PE-NPs alone.

### 3.4. In Silico Docking Study

#### 3.4.1. Effect of PE-NPs on Inflammatory Mediators

**Figure 7:**
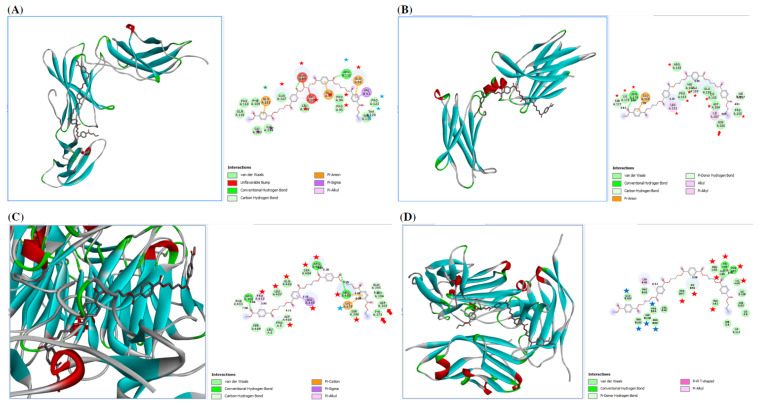
PE-NPs docked to IL-6R via binding to Pro94, Pro95, Glu96, Glu97, Gln99 (red asterisk), Arg118, Ser119, Thr120, Pro121 (blue asterisk), Gly193 and Ile194 (violet asterisks), and Glu283 (green asterisks) (**A**); IL-17R via binding to Ser177, Lys178, Asn179, Leu181, Pro183, Glu186, His187, Ala188, Arg189, Lys191, Asp204, Pr205, Asn206, Ile207 (red asterisks) (**B**); integrin CD41/CD61 via binding to many amin acids especially, Asp369 and Tyr371 (red arrow), Ser396, Arg400, Arg402, Ser404, Gln405, Val406, Leu407, Asp408, Pro410 (red asterisk) and Lys678 (blue asterisk) (**C**); and CD47/SIRP via binding to amino acids located within the highest predicted B-cell epitopes on SIRP (Gly55, His56, Val60, Thr61) of chain A and (His56 and Phe57) of chain B (red asterisk) followed by (Phe94, Pro99, Asp100, Thr101, Glu102) of chain B (blue asterisk) (**D**).

The in silico results are shown in Figure 7. PE-NPs docked to IL-6R by binding to many residues located within the B-cell epitope on IL-6R. Also, PE-NPs docked to IL-17R by binding to many residues, especially Asn206, the N-glycosylation site, and to critical residues within the highly predicted B-cell epitopes on IL-17R.

PE-NPs docked to integrin aIIBb3 (CD41/CD61) by binding to many amino acids, especially Asp369 and Tyr371, which are located within the binding site of the metal-ion-dependent adhesion site (MIDAS), as well as many residues, such as Ser396, Arg400, Arg402, Ser404, Gln405, Leu407, Asp408, Pro410, and Lys 648, which lie within the B-cell epitope of alpha 2b subunit.

PE-NPs docked to CD47/SIRP by binding to critical amino acids in SIRP, including Gly 55, His56, Phe57, Pro58, Thr101, and Glu102. Additionally, PE-NPs bound to the highly predicted B-cell epitopes on SIRP by binding to amino acids within the highest predicted B-cell epitopes, including Gly55, His56, Phe57, Val60, and Thr61, followed by Phe94, Pro99, Asp100, Thr101, and Glu102.

#### 3.4.2. Effect of PE-NPs on the Cell Cycle

**Figure 8:**
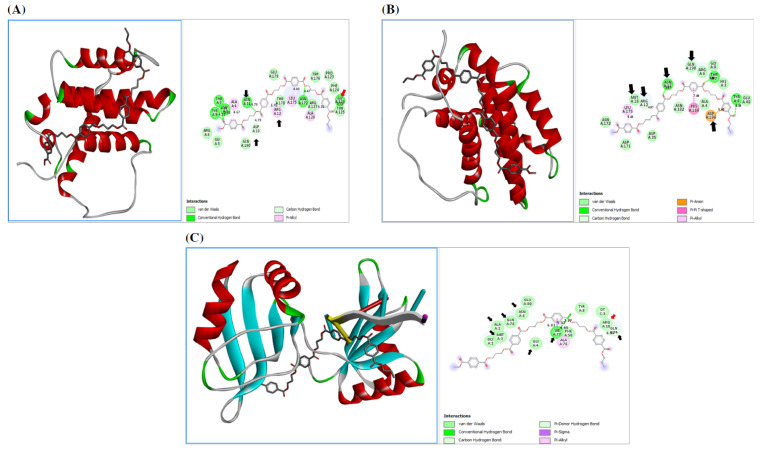
(**A**) Docking of PE-NPs to BCL-2 isoform 1 by binding to many residues, especially those located within the BH1 and BH4 apoptosis regulator signature (black arrow); (**B**) Docking of PE-NPs to BCL-2 isoform 2 by binding to many residues, especially those located within the BH4 apoptosis regulator signature (black arrow) and site of phosphorylation by MAPK (red arrow); (**C**) Docking of PE-NPs to c-MYC by binding to many residues, especially those located within the sulfate binding sites (black arrow) and nucleotide DT (red arrow).

Figure 8 shows that PE-NPs docked to c-MYC (6m75) by binding to many residues, especially Arg10, Gln74, Lys77, and Gln78. PE-NPs also bound to many residues on IL-17R, especially Asn206. PE-NPs docked to BCL-2 isoform I (1g5m) by binding to many residues, especially critical ones, such as Gln190 and Asp196, as well as Asn11, Arg12, and Met16, which are located within the BH2 and BH4 motif signatures, respectively. Also, PE-NPs docked to BCL-2 isoform II (1gjh) by binding to many residues, especially critical ones, such as Asp10, Asn11, and Arg12, which are located within the BH2, as well as Gly128.

#### 3.4.3. Protective Effect of Biobran Against PE-NPs Toxicity

**Figure 9:**
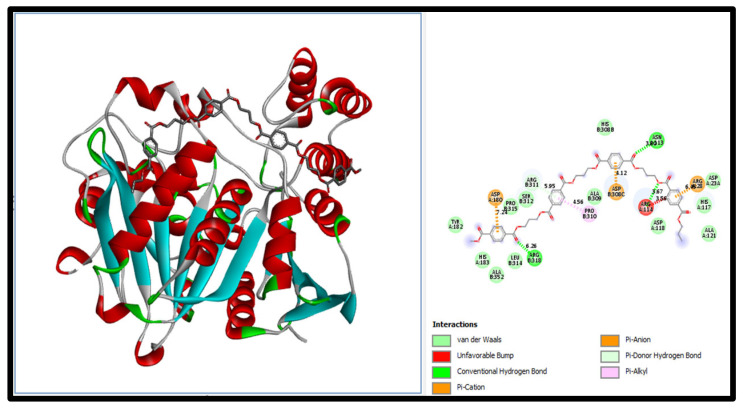
Serine carboxypeptidase docking to PE-NPs.

The docking of PE-NPs to serine carboxypeptidase, the active ingredient of Biobran, is shown in Figure 9. The binding energy between serine carboxypeptidase and PE-NPs was −145.0 Kcal/Mol, which was lower than that between PE-NPs and the other examined proteins for both pro-inflammatory mediators (IL-6R, IL-17R, CD47/SIRP) and oncogenic proteins (c-MYC and BCL-2 isoform I and BCL-2 isoform II), where the binding energies were (−118.27, −119.8, −135.41 Kcal/Mol) and (−103.66, −123.33, −135.34 Kcal/Mol), respectively. Thus, the affinity of carboxy peptidase to PE-NPs was higher than that between PE-NPs and both pro-inflammatory mediators and oncogenic proteins.

## 4. Discussion

The results of the current study demonstrate the protective effects of Biobran/MGN-3, an arabinoxylan from rice bran, against the cytotoxic effects of polyethylene nanoplastics (PE-NPs) for normal mouse hepatocytes. PE-NPs had cytotoxic effects on liver cells in a concentration-dependent manner, with hepatotoxicity associated with pre-G1 and G2/M cell phase arrest and apoptotic cell death induction. These results are consistent with other studies that have reported the toxic effects of NPs in various other cell types (including lymphocytes, intestinal cells, testicular cells, osteoblasts, and lung cells) and have consistently shown that exposure to plastics resulted in a decrease in cell viability and raised cytotoxicity [43,44,45,46]. More importantly, we demonstrate for the first time that Biobran could alleviate the hepatotoxicity induced by exposure to PE-NPs. Prior studies have shown the ability of Biobran to overcome liver toxicity in different models, including animal studies and patients with hepatocellular carcinoma. Animal studies have revealed the protective effects of Biobran against N-nitrosodiethylamine (NDEA) and carbon tetrachloride CCl_4_-induced hepatocarcinogenesis in rats [47]. Treatment with Biobran prevented body weight loss and an increase in liver weight caused by NDEA. In addition, Biobran treatment induced a remarkable increase in liver function enzymes, including aspartate aminotransferase (AST), alanine aminotransferase (ALT), alkaline phosphatase (ALP), and γ-glutamyl transpeptidase (γ-GT) in animals exposed to carcinogens [47]. Furthermore, our studies revealed the possible mechanisms by which Biobran inhibits hepatocarcinogenesis in rats. These include induction of apoptosis, inhibition of inflammation, and suppression of cancer cell proliferation, suggesting Biobran’s promising chemopreventive and chemotherapeutic use against liver carcinogenesis [48]. Furthermore, our earlier clinical trial of adult patients with hepatocellular carcinoma showed that the addition of Biobran to interventional therapies, including transarterial chemoembolization, percutaneous ethanol injection, radiofrequency ablation, and cryoablation, resulted in improved overall survival, reduced tumor size, less recurrence of cancer, and a lower alpha-fetoprotein level [25]. In contrast, other biological response modifiers (BRMs), including PSK, lentinan, and OK-432, showed no effect when combined with 5-FU for the treatment of HCC [49].

Earlier studies have also shown Biobran to be a potent antioxidant agent as manifested by its ability to improve antioxygenic potential and protect against oxidative stress in mice [50]. This characteristic could be attributed to Biobran’s augmentative effect on macrophage phagocytosis [51], as well as its ability to enhance the intracellular killing of microbes by human phagocytic cells in vitro [52]. Recent studies showed that Biobran can protect against streptozotocin-induced sporadic Alzheimer’s disease (SAD) through modulation of the apoptotic and oxidative stress pathways. This includes Biobran’s ability to reverse the spatial memory deficit in SAD-induced mice; increase the expression of glutathione (GSH); reduce malondialdehyde, IL-6, and ICAM-1; suppress caspase-3 and the pro-apoptotic protein Bax; and upregulate the anti-apoptotic protein BCL-2 [53]. The antioxidant potential of Biobran was also noticed in another model of female albino mice that were inoculated intramuscularly with Ehrlich ascites carcinoma (EAC) cells, followed by Biobran IP injection. Biobran treatment enhanced antioxidant potential by modulating lipid peroxidation (LPx), augmenting GSH contents, and upregulating the expression of glutathione peroxidase (GPx), superoxide dismutase (SOD), and catalase (CAT) [50]. Biobran has thus been found to induce oncostatic activity by modulating lipid peroxidation, augmenting the antioxidant defense system, and protecting against oxidative stress.

Further studies have reported the protective effects of Biobran on radiation-induced intestinal injury by a mechanism that reduces the oxidative stress levels and inflammatory response indicators in the serum, as well as in the jejunal and colonic mucosa. Exposure to Biobran causes a significant increase in antioxidant indicators such as SOD, GPx, and CAT, as well as total antioxidant capacity. In addition, Biobran decreases the gene abundances and enzymatic activities of caspase-3, -8, -9, and -10 [54]. Other studies have shown that Biobran treatment significantly protects against D-GalN-induced liver injury and hepatitis in rats [55]. Low-molecular-weight fractions of Biobran also exert protective effects against acute liver injury by inhibiting the expression of JNK/MAPK and NF-кB [56]. The beneficial effects of Biobran in antioxidation, hepatoprotection, antiinflammation, immunomodulation, and synergistic anticancer properties have been well illustrated in a recently published review article [57].

In the current study, we measured PE-NPs and Biobran’s effect on the cell cycle of liver cells. The cell cycle is a cornerstone of cellular vitality [58], and cell cycle dysregulation is an essential aspect of the toxicity of NPs to normal cells [59]. Earlier studies demonstrated the G0/G1 cell cycle arrest phase in NIH 3T3 cells following NP treatment [60]. We found that PE-NPs induced pre-G1 and G2/m arrest, highlighting the hepatotoxic effect of NPs in inducing cell death and interfering with the mitosis process in normal liver cells, which may lead to cellular senescence [61]. Antioxidants play an important role in inhibiting the activity of scavenging radicals, thus protecting humans against infection and degenerative diseases. Biobran is an arabinoxylan with an arabinose polymer in its side chain and xylose in its main chain [26], and it has been extensively studied for its various biological activities, including anticancer, anti-inflammatory, and antioxidant properties [48]. In an animal study, liver cancer was induced by a carcinogen (NDEA plus CCl4) [25]. Subsequently, molecular studies of gene and protein expression and DNA fragmentation were carried out [48]. Treatment with Biobran resulted in the following: (1) the arrest of cancer cells in the sub-G1 phase of the cell cycle; (2) increased DNA fragmentation in cancer cells; and (3) marked upregulation for p53, Bax, and caspase-3 gene expression, as well as marked downregulation in Bcl-2 gene expression compared to the untreated carcinogen group. Furthermore, Biobran treatment increased the Bax/Bcl-2 ratio and caspase-3 expression compared to the untreated carcinogen group. This is an important finding that has been considered by others as a mechanism for preventing hepatocarcinoma [62,63,64].

Finally, the immunomodulatory effects of Biobran on NK cells and cytokine production may represent a third mechanism where this agent is able to generate a protective effect against the cytotoxic effects of PE-NPs. The effects of NPs on the immune system have been the focus of many researchers. The results of these studies show that the interaction between the innate immune system and NPs depends on factors that include the NP size and shape. For example, silica–titania hollow nanoparticles with a 50 nm diameter generated more ROS and exhibited the highest induction of inflammatory cytokines (IL-1, IL-6, and TNF-α) with mouse alveolar macrophages in vitro [65]. In addition, hydrophobicity and surface modification are other major factors that influence the interactions between NPs and the innate immune system [66]. NPs can communicate with various biological components (cells, receptors, proteins) of the immune system, trigger cell signaling cascades, and consequently cause unpredictable immune responses (activation or suppression) and even harmful outcomes (autoimmune diseases or cancer) [67,68,69]. Biobran has been studied extensively for its immunomodulatory effects on NK cells and cytokine production. NK cells play an important role in immune surveillance against cancer [52,70,71] and have been shown to kill target cells, including tumor cells, via different pathways, such as the ligation of FasL to its Fas receptor, to induce apoptosis [72,73,74,75]. Our earlier studies have demonstrated activation of NK cell activity following exposure to oral administration of Biobran in normal humans [26,27,34] and cancer patients [33], as well as IP administration of Biobran in aged mice [76]. The protective effects of Biobran against the cytotoxic effects of PE-NPs for normal mouse hepatocytes may involve mechanisms that include its antioxidant and apoptotic capabilities, as well as its immunomodulatory effects. The data may establish the foundation for in vivo studies that could have therapeutic implications.

Our study further complements the experimental findings with an in silico study. The first finding was that PE-NPs can interact with pro-inflammatory mediators, including IL-6R, IL-17R, CD41/CD61, and CD47/SIRP, and with oncogenic proteins, including c-MYC and BCL-2. Based on the data published about the structure of the examined proteins, we detected the critical residues for each. The inflammatory effects of PE-NPs were noticed through their interactions with IL-6R and IL-17R. In particular, PE-NPs targeted the N-glycosylation site (Asn206) of IL-17R and may, thus, destabilize the structure of IL-17R. It has been demonstrated that the N-glycosylation of Asn, together with the disulfide bonds between cysteine residues and methionine, maintains the architecture of IL-17R. The effect of PE-NPs on inflammatory mediators was clearly observed; it can interact with integrin CD41/CD61 by targeting Asp369 and Tyr371, which are located within the binding site of the metal-ion-dependent adhesion site (MIDAS), as reported by Xiao et al. [77]. We may propose a mechanism for PE-NP-induced inflammation involving the disruption of integrin CD41/CD61 binding to its ligand, causing platelet disorders. Additionally, the binding of PE-NPs to CD47/SIRP at highly predicted B-cell epitopes on SIRP, including Gly 55, His56, Phe57, Pro58, Val60, Thr61, Thr101, and Glu102 on SIRP, may represent another mechanism of inflammation by stimulating B cells to produce antibodies.

The in silico results also provide evidence for the effect of PE-NPs on the cell cycle. PE-NPs dock to BCL-2 isoform I by binding to many residues, which include critical ones lying within the BH2 and BH4 motif signatures that regulate apoptosis. Binding PE-NPs to BCL-2 at the signature motif may enhance the anti-apoptotic signal, while phosphorylation of BCL-2 on Gly inhibits the anti-apoptotic action of BCL-2. In addition to the ability of PE-NPs to bind to the BH2 motif signature, they can bind to one of the phosphorylation sites. As reported, phosphorylation of BCL-2 inhibits its anti-apoptotic action. Thus, the binding of PE-NPs to the BH2 and BH4 domains of BCL-2 may inhibit its anti-apoptotic effect, as reported by Reed et al. [78], who demonstrated that the BH2 or BH4 domains of BCL-2 abolish its ability to suppress cell death. Therefore, we suggest that PE-NPs can promote hepatocytotoxicity by enhancing the cell death of liver cells. Another important result is that PE-NPs interact with c-MYC at the sulfonation site since it has been found that sulfate enhances the cell transformation by c-MYC and alters cell apoptosis [79]. Collectively, we may suggest that PE-NPs induce hepatotoxicity by both inhibiting the anti-apoptotic effect of BCL-2 and modulating the proliferative effect of c-MYC.

Our data further support the efficacy of Biobran in counteracting PE-NP-induced liver toxicity and oncogenicity, as our findings show that Biobran’s affinity for PE-NPs overcomes the affinity of PE-NPs for all examined inflammatory mediators and cell cycle regulators. The main finding of our in silico study is that serine carboxypeptidase, an active ingredient in Biobran, can capture and block all nucleophilic centers of PE-NPs. The carcinogenicity of nucleophilic compounds such as the benzene ring and ethylene derivatives has been well established [80,81,82]. Our data suggests a novel anti-carcinogenicity effect of Biobran via scavenging the nucleophilic effect of PE-NPs.

To the best of our knowledge, no prior study has directly tested the protective effects of Biobran against nanoparticle-induced liver toxicity. We found that Biobran effectively countered the toxicity of NPs, as exemplified by its ability to restore cell viability to a level comparable to the control group. The mechanisms include the reversal of nanoparticle-induced alterations in cell cycle phases and the reduction of apoptotic cell death induced by nanoplastics.

## 5. Conclusions

Biobran, an arabinoxylan from rice bran, has been shown to protect normal mouse hepatocytes against the cytotoxic effects of polyethylene nanoplastics. The viability of liver cells was significantly improved by Biobran through a mechanism involving apoptotic effects. These findings suggest that Biobran holds promise as a protective agent against nanoplastic-induced toxicity and provide hope for counteracting the notorious effects of these pervasive particles on human well-being and longevity.

## Data Availability

Data from the current study are available from the corresponding author upon reasonable request.
